# Concurrent, Performance-Based Methodology for Increasing the Accuracy and Certainty of Short-Term Neural Prediction Systems

**DOI:** 10.1155/2019/9323482

**Published:** 2019-04-01

**Authors:** Miljana Milić, Jelena Milojković, Ivan Marković, Petar Nikolić

**Affiliations:** ^1^Faculty of Electronic Engineering, University of Niš, Aleksandra Medvedeva 14, 18000 Niš, Serbia; ^2^Innovation Centre of Advanced Technologies, Bulevar Nikole Tesle 61, Loc. 5, 18000 Niš, Serbia; ^3^Faculty of Economics, University of Niš, Trg Kralja Aleksandra Ujedinitelja 11, 18000 Niš, Serbia; ^4^Tigar Tyres, Nikole Pašića 213, 18300 Pirot, Serbia

## Abstract

Accurate prediction of the short time series with highly irregular behavior is a challenging task found in many areas of modern science. Such data fluctuations are not systematic and hardly predictable. In recent years, artificial neural networks have widely been exploited for those purposes. Although it is possible to model nonlinear behavior of short time series by using ANNs, very often they are not able to handle all events equally well. Therefore, alternative approaches have to be applied. In this study, a new, concurrent, performance-based methodology that combines best ANN topologies in order to decrease the forecasting errors and increase the forecasting certainty is proposed. The proposed approach is verified on three different data sets: the Serbian Gross National Income time series, the municipal traffic flow for a particular observation point, and the daily electric load consumption time series. It is shown that the method can significantly increase the forecasting accuracy of the individual networks, regardless of their topologies, which makes the methodology more applicable. For quantitative comparison of the accuracy of the proposed methodology with that of similar methodologies, a series of additional forecasting experiments that include a state-of-the-art ARIMA modelling and a combination of ANN and linear regression forecasting have been conducted.

## 1. Introduction

Prediction is a process that uses data from the present and the past in order to estimate future. The result of this process is the information about probable events in the future and their effects and outcomes. Making good forecasts is essential for making good decisions and planning in all areas of life. Although it does not have to reduce uncertainties and difficulties of the future, it can increase the certainty and the level of the preparedness for challenges and environmental changes that future events bring.

The need for development of prediction methods occurs in almost every area of life—technology, engineering, industry, science, politics, economy, business, sport, medicine, etc. Good forecasts can ensure lower cost of the services and products, increased customer/client satisfaction, and significant competitive advantage [1].

Every daily activity begins with planning. The planning begins with a prediction [2]. Prediction errors may have crucial implications on decision-making, profits and investment justification, risk assessment, alerting events, hard real-time systems' actions, timely handling of emergency health and medical conditions, etc. [3]. Because of that, decreasing the error of the prediction is an essential task for every forecasting expert, regardless of the applied prediction methods.

Prediction methods described in the literature can be roughly categorized into two large groups: traditional and modern. Each of them has advantages and disadvantages. None of them is superior to all others if we consider all possible criteria of evaluation [4]. Traditional prediction methods try to extrapolate time series data using different modelling: exponential smoothing [5], linear or nonlinear regression [6–8], simple (AR) or more complex autoregressive models (ARMA, ARIMA, and double seasonal ARIMA) [6, 9]. On the other hand, modern prediction methods exploiting the artificial intelligence (AI) behavior can model both nonlinear and linear structures of time series [10] and can produce good accuracy of the forecasting. Such techniques use different topologies of artificial neural network, fuzzy modelling, vector machines, and genetic-simulated annealing algorithms to predict time series data [4, 11–14]. Different authors have shown that AI-based models frequently express better predictive characteristics compared to models using standard multilinear regression [14]. Finally, both theoretical and empirical findings in the literature show that combination of two or more different methods can be an effective and efficient way to improve forecasts and decrease the error [10]. Such hybrid methods are studied in [2, 3, 10, 15–17].

Despite numerous ways to predict the future mathematically, there are many cases of variables that could not reliably be predicted. Causes for this limitation could be found in the randomness of the events and the lack of significant relationship in data. When factors considered during forecasting of a certain variable are not well known or understood, prediction becomes imprecise or mistaken. Sometimes, there is simply not enough data about everything that affects the forecasted variable. The prediction process relies on some specific hypothesis. If they are set wrong, due to bad judgment, i.e., human error, the prediction will be mistaken. Although the forecasting is based on past events, no one can guarantee that the history will repeat every time in the same way. Therefore, forecasts are subject to human error.

A time series can be defined as a sequence of numerical data occurring in regular intervals over a period of time, collected in a successive order. Short time series are characterized by a lack of trend information, randomness and periodicity, and demands for such forecasting represent a challenging problem [18]. Usually, time series cases where the sample length *N* is very small are not applicable for generating statistically reliable variants of forecasting. In this paper, we will focus on such time series and their forecasting. We will propose a new methodology that can be applied to irregular series. The methodology is applicable to all types and topologies of neural networks, or similar AI based forecasting methods, in order to improve their accuracy.

The usual step in development of forecasting ANN is to train many networks, while changing the number of neurons in some particular layer. The ANN with the most accurate forecasting wins. Nevertheless, if we observe the forecasts of all obtained networks, we can conclude that sometimes different networks predict different directions of the trend change in the next forecasting step. In this point, one cannot determine which one is the correct. This is particularly noticeable when dealing with volatile data series. Therefore, incorporating more than one network in the forecasting decision could make better predictions of the future events. The methodology that is suggested in this paper improves the forecasting accuracy of the ANN in the sense that it concurrently exploits several most accurate networks instead of the winner one. In this way, the forecasting accuracy can be significantly improved, as well as the confidence of the prediction. The performance of the proposed method is verified on an example of Serbian Gross National Income (GNI) data series, using Feed Forward Accommodated for Prediction (FFAP) neural networks' topology. The results demonstrate higher forecasting accuracy compared to individual FFAP networks.

The rest of the paper is organized in the following manner. In Section 2, the structure of the FFAP neural network topology is presented. The section that follows describes in detail the concurrent best-performance-based methodology for increasing the accuracy of the short time series FFAP forecasting. Three case studies are performed and analyzed: Serbian Gross National Income time series, the municipal traffic flow for a particular observation point, and the daily based electric load consumption time series; the forecasting results of the proposed methodology and other state-of-the-art forecasting techniques and their combinations are given in Section 4. The obtained results are discussed in Section 5, while conclusions are summarized in the last section.

## 2. FFAP Neural Networks

In general, neural network-based computational and forecasting methods developed from the desire to reveal, realize, and emulate the capability of the brain to process information [14]. The entire brain is composed of many neural networks that receive information from the surroundings, extract and recombine their relevant parts, and make the decisions about the needs of the organism. Artificial neural networks (ANN) emulate such abilities of the brain in order to realize complex nonlinear input-output transformations.

Consider a time series denoted by *y*_*i*_, *i*=1, 2,…, *m*. It is a set of observables of an undefined function $\hat{y}=\hat{f}\left(t\right)$, that are taken at regular time intervals Δ*t*, where *t*_*i*+1_=*t*_*i*_+Δ*t*. In the forecasting process, the historical data are used to determine the direction of future trends, while one-step-ahead forecasting implies the mathematical search for such a function *f* that can accurately perform the following mapping:

(1) $$\begin{array}{c} y_{m+1}=f\left(t_{m+1}\right)={\hat{y}}_{m+1}+\varepsilon , \end{array}$$

where the term ${\hat{y}}_{m+1}$ represents the desired response, while *ε* is the acceptable error.

In the past decades, ANNs have been developed as a tool that has great capabilities for recognizing and modelling data patterns that are not easily identifiable by traditional methods. However, one may notice a common feature in all existing ANN applications in forecasting. It is the necessity for a relatively long time series in order to achieve high accuracy. Usually, there should be at least 50 data points to consider [19]. Because of this and due to previous research in short-term forecasting [20–22], we have chosen the FFAP neural network topology, as a base to be used throughout this study. This structure will briefly be explained next.

**Figure 1:**
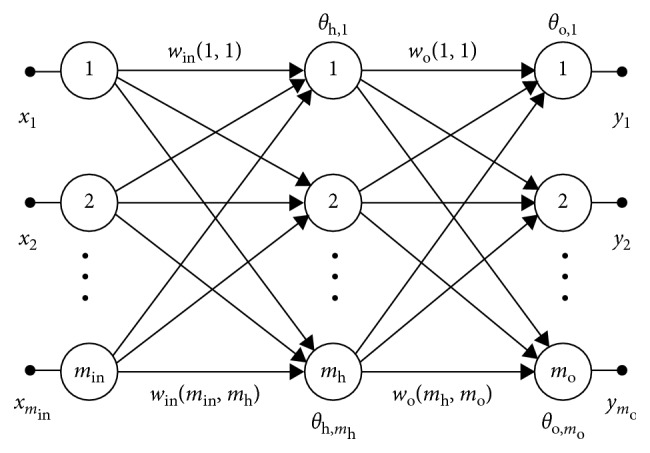
Basic fully connected feed forward neural network (one hidden layer and multiple outputs in the output layer).

General structure of a feed forward neural network is illustrated in Figure 1. It has just one hidden layer, since it is confirmed to be sufficient enough to solve univariate forecasting problems [23]. In this figure, indices “in,” “h,” and “o” denote input, hidden, and output layers of the ANN, respectively. Weights are labeled with *w*(*k*, *l*), where connections of the input and the hidden layer are designated with *k* = 1, 2,…, *m*_in_, *l* *=* 1, 2,…, *m*_h_, while connections of the hidden and output layer are designated with *k* *=* 1, 2,…, *m*_h_, *l* *=* 1, 2,…, *m*_o_. The thresholds are denoted with *θ*_x,r_, *r* *=* 1, 2,…, *m*_h_ or *m*_o_, depending on the layer. The neurons in the input layer distribute the input signals, while neurons in the hidden layer are activated by a sigmoid function. Finally, linear function activates neurons in the output layer. A modified version of the steepest-descent minimization algorithm is applied as a learning method [24]. The problem of initialization was solved using the method described in [25].

In the case of short time series prediction problem, a set of observables (samples) is given (per time instant) meaning that only one input signal is available, the discretized time [18]. To enable mathematical solution for the forecasting problem, in most cases, both values for time variable and the response need to be transformed, as shown in the following equation:

(2) $$\begin{array}{c} t=t^{*}-t_{0}. \end{array}$$

Having in mind that *t* ^*∗*^ stands for the time instant, this reduction gives the value of 1 to the time of the first sample (*t*_0_). Samples are normalized in the following way:

(3) $$\begin{array}{c} y=y^{*}-M, \end{array}$$

where *y* ^*∗*^ stands for the current value of the target function and *M* is a constant, selected so as to reduce the relative difference between output values, if necessary. When implementing the architecture in Figure 1, the following series would have to be learned: (*t*_*i*_, *f*(*t*_*i*_)), *i* = 1,…, *m*.

**Figure 2:**
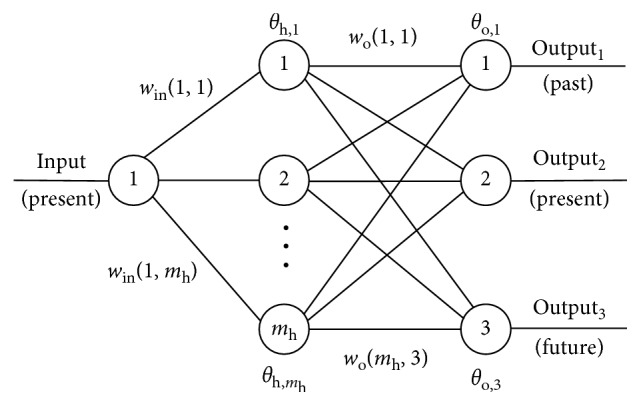
FFAP—feed forward accommodated for prediction ANN structure.

Exploiting the basic topology shown in Figure 1, in [26, 27], better forecasting solutions were suggested for the problem of short prediction base period. This architecture is referred to as feed forward accommodated for prediction (FFAP) and depicted in Figure 2. The main idea during the FFAP architecture development was to force the neural network to learn the same mapping several times simultaneously but shifted in time. In that way, it is supposed that previous responses of the function will have larger influence on the *f*(*t*) mapping. This also forces the network to identify complex intertwined deterministic relations existing between phenomena that influence the observed variable.

The FFAP architecture is depicted in Figure 2. The input set (*t*_*i*_) is brought to the input terminal. The future terminal at the *Output*_3_, approximates *y*_*i*+1_. *Output*_3_ may also be seen as a vector when a multiple-step prediction is required. The present value *y*_*i*_ is obtained at terminal *Output*_2_. Finally, *Output*_1_ should learn the past value, i.e., *y*_*i*-1_. Although it is not explicitly stressed out, this may also be seen as a vector of past values of the response (since multiple samples from the past responses were used).

We can express the functionality of the network as

(4) $$\begin{array}{c} \left(y_{i+1},y_{i},y_{i-1},y_{i-2},y_{i-3}\right)=f\left(t_{i}\right),\;i=4,...,m, \end{array}$$

where Output_1_={*y*_*i*−1_, *y*_*i*−2_, *y*_*i*−3_}, meaning that one future, one present, and three previous responses are to be learned.

Our task here was to do one-step-ahead prediction. Using the already predicted value as input data for multistep-ahead prediction leads to accumulation of the prediction error what we demonstrated in [21].

In this way, an efficient network topology is created, that uses in the test phase, only matrix *w*_in_ in combination with single column/row of matrix *w*_o_, and the outputs corresponding to different moments, for evaluation of weighs in *w*_in_ and thresholds in hidden layer.

## 3. Concurrent Best-Performance-Based Prediction Methodology

**Figure 3:**
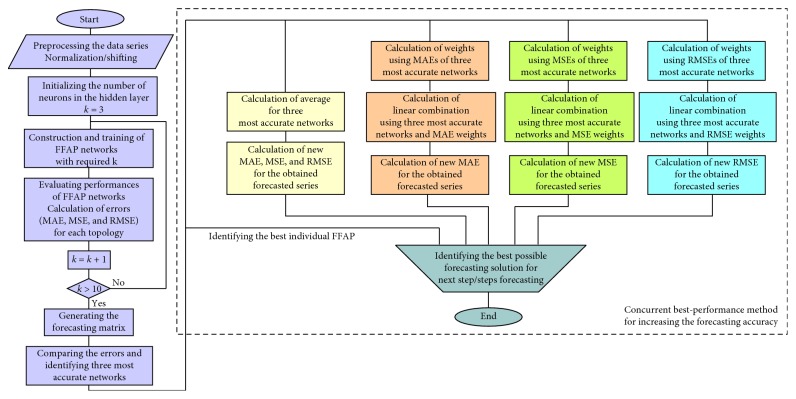
Algorithm for obtaining high-accuracy predictions.

The methodology for increasing the accuracy and certainty of short time series ANN forecasting that we are suggesting is depicted in Figure 3. The aim of this procedure is to establish tools and procedure that will increase the accuracy of the existing individual forecasting FFAP networks, exploiting the best of them, in a concurrent manner.

We start with a turbulent short time series, expressing a certain variable for a given period of time. In order to facilitate their learning, these data sets have to be properly adjusted (shifted and/or normalized). After that, we evaluate the effect of changing the number of neurons in the hidden layer of the FFAP on the forecast accuracy. We begin with generating and training the FFAP network that has 3 neurons in the hidden layer. The experiment repeats with increasing number of hidden neurons. The construction of networks ends when FFAP with 10 neurons in the hidden layer is obtained and trained. This gives 8 different neural networks.

When a single network is trained, it requires a minimal learning set of first 13 samples (in our case, this is shown to be enough) in order to create the first forecast at the output, that is the extrapolated value of the trend curve. Since this is still training, one can also calculate the first forecasting error for the particular network. By entering every further sample, the network can better learn and predict the trend and its change for the input variable. As already mentioned, this process repeats for the entire group of networks. At the end of this process, all networks are trained with the entire time series, representing the past and the present. The result of this process can be systematized in a form of a forecasting matrix, with columns representing the number of neurons in a particular network, while rows represent time instances for which the forecast were made. The number of columns goes from three to ten, while number of rows is equal to the number of input samples.

In the next step, we analyze the forecasting matrix and search for three topologies that have the best performance measures. The reason for choosing three topologies lies in the fact that the time series can be irregular. Two best networks can predict totally opposite trend change for the next predicting step.

Because no one can tell which one is more correct, we introduce the forecast of the third best network to perform a kind of arbitration. In this way, the confidence of the prediction as well as its accuracy can be improved. In this analysis, we calculate mean average error (MAE), mean square error (MSE), root-mean-squared error (RMSE), and mean absolute percentage error (MAPE), for each column, using equations (5)–(8). The least three values of a particular error correspond to three most accurate ANN forecasting topologies:

(5) $$\begin{array}{c} \text{MAE}=\frac{\sum_{i=1}^{N}\left|y_{i}-{\hat{y}}_{i}\right|}{N}, \end{array}$$

(6) $$\begin{array}{c} \text{MSE}=\frac{\sum_{i=1}^{N}{\left(y_{i}-{\hat{y}}_{i}\right)}^{2}}{N}, \end{array}$$

(7) $$\begin{array}{c} \text{RMSE}=\sqrt{\frac{\sum_{i=1}^{N}{\left(y_{i}-{\hat{y}}_{i}\right)}^{2}}{N}}, \end{array}$$

(8) $$\begin{array}{c} \text{MAPE}=100\cdot \frac{\sum_{i=1}^{N}\left|\left(y_{i}-{\hat{y}}_{i}\right)/y_{i}\right|}{N}, \end{array}$$

where *N* denotes the number of sample cases, *y*_*i*_ represents the individual predicted value, and *^* ${\hat{y}}_{i}$ is the target value for the sample *i*.

The process ends by adopting the final prediction on a test set data calculated using three best topologies simultaneously. Four cases of concurrent forecasting were investigated. First, we simply calculate an average of the forecasts for three most accurate networks. Namely, if the two of three predictions are supporting each other in forecasting the future trend, they make the largest impact to the final prediction, decreasing the importance of the third that predicts the opposite change of the trend. In that way, none may be qualified as the better one. So, the average is the best representative.

Nevertheless, from our experience, creating a linear combination of three best forecasts, where weights or a multiplying factor for each network, corresponds to a reciprocal of its forecasting error, can further improve the forecasting accuracy. The one that was the closest to the correct value gets the largest weight in the equation for the final forecast, as shown in the following equations:

(9) $$\begin{array}{c} y_{\text{final}}=w_{1}\cdot y_{\text{f}1}+w_{2}\cdot y_{\text{f}2}+w_{3}\cdot y_{\text{f}3},\\ w_{1}:w_{2}:w_{3}=\varepsilon_{3}:\varepsilon_{2}:\varepsilon_{1},\\ w_{1}+w_{2}+w_{3}=100\%, \end{array}$$

where *ε* can be of any error types that are calculated (MAE, MSE, or RMSE). In these equations *y*_final_ denotes the final forecast value, while *y*_f1_, *ε*_1_; *y*_f2_, *ε*_2_; and *y*_f3_, *ε*_3_ denote forecasts of the first, second, and third most accurate networks and their corresponding errors, respectively.

## 4. Case Studies

### 4.1. Prediction of the Serbian Gross National Income

Gross National Income (GNI) is defined as an estimate of the income from goods and services produced by an economy and received by a country both domestically and from abroad. This measure of the size of an economy is highly important and has large economic, political, and social implications. For politically and economically turbulent developing countries, it is very difficult to predict it due to mutual influence of many known or unknown factors. The methodology that can be considered for such predictions exploits artificial neural networks. This kind of time series appears superb for verifying the methodology that we propose. In this study, we will try enhancing the neural network approaches, described in Section 2, for short -term forecasting of the Serbian GNI, by decreasing the forecasting error and increasing the confidents of the predictions in the next term, using its historical data. The same data set will be used for ARIMA forecasting in order to create a feeling about the order of accuracy of the proposed methodology.

For the analyzed series, time is discretized at year long periods and reduced by 1989, as given by (2), while the value of the GNI is given in billions of Dollars, like in original data. In this case, there was no need for normalization of data. However, in our experience, these transformations can make the training process numerically better conditioned.

Although this time series covers a long period of time (1990–2017), the GNI is the economic variable that is obtained annually. It was first introduced by economic scientists in 1990s. Because of that, this time series can be considered as a very short. The time series data are obtained from the World Bank National Accounts data and OECD National Accounts data files.

**Figure 4:**
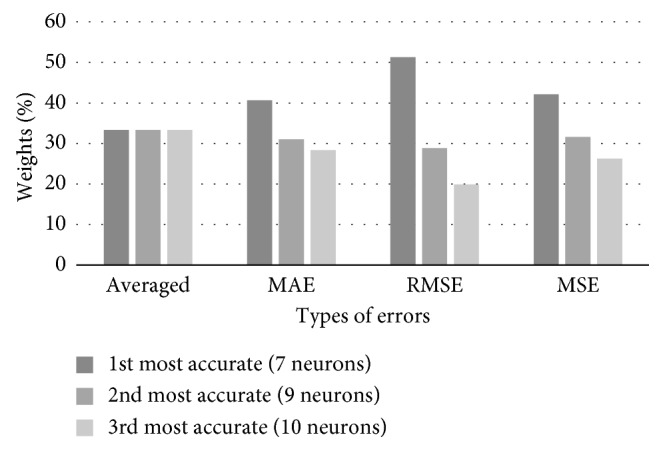
Combined weights of three best networks predicting GNI, corresponding to particular error types.

After the initial training of 8 networks using the test set that contains GNI data for a period 1990–2012, we have calculated their performance measures, i.e., MAE, RMSE, and MSE. Three most accurate FFAP networks have 7, 9, and 10 neurons in the hidden layer. Corresponding weights were calculated based on the values of the networks' errors in order to be used in combined predictions of the test set (2013–2017). Values are listed in Table 1, while Figure 4 gives their graphical representation.

**Figure 5:**
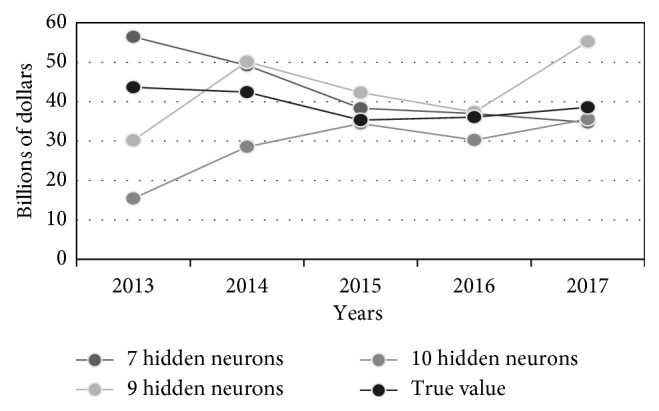
GNI forecasting of three most accurate FFAP ANNs.

**Figure 6:**
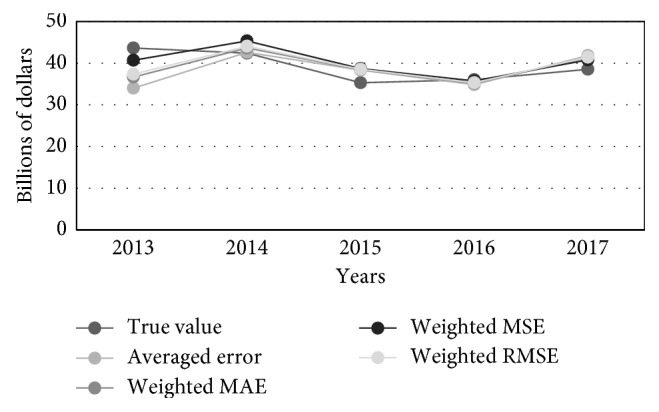
Combined predictions of the GNI: true, averaged, MAE-weighted, RMSE-weighted, and MSE-weighted values.

Table 2 contains prediction results obtained for 5 successive time instants of the test set, starting from 2013, as well as the true value of the GNI.  Figure 5 visualizes three best GNI predictions from Table 2, while Figure 6 illustrates different GNI predictions, i.e., true, averaged, MAE-weighted, RMSE-weighted, and MSE-weighted values.

As a final measure of performances, for the proposed methodology, we have calculated MAE, MSE, RMSE, and MAPE for all GNI predictions of the test set for the period 2013–2017, and these are shown in Table 3. All types of errors obtained after applying our forecasting algorithm are better than any particular FFAP. For example, the best FFAP that has 7 neurons in the hidden layer had a RMSE for the entire predicted period of 6.85, while the best improvement in forecasting is achieved when MSE-weighted concurrent linear combination of three best FFAP is applied. RMSE in this case is 2.60. The error reduction in this case is almost 65%.

An economic variable that is very similar to the GNI is the GNI per capita. It is an economy measure that is obtained when the value of the GNI is divided by the number of residents in a country. It should be emphasized that values for the GNI and the GNI per capita show very similar trends for particular time interval in the case of the Republic of Serbia. This could be explained by the fact that the natality for the Republic of Serbia is varying very slow over time. The authors have performed the similar forecasting procedure on a GNI per capita time series for the same time period, and it has shown very similar forecasting performance measures.

To the best of our knowledge, analyzing GNI time series in the case of Serbian economy is the task that has not been studied or published yet, and no comparative analysis of the forecasting accuracies with some alternative forecasting methods can be conducted. To overcome this problem alternative state-of-the-art, i.e., autoregressive integrated moving average forecasting methodology (ARIMA) was performed on the same data set. The theoretical background on this topic as well as its implementation strategies can be found in the literature [28, 29].

The model fitting process for GNI training set will be very briefly explained here, since this task is out of the scope of this study. To achieve the adequate ARIMA(*p, d, q*) model, GNI series was tested for stationarity by applying the unit root tests: Kwiatkowski–Phillips–Schmidt–Shin (KPSS) test and augmented Dickey–Fuller test (ADF). They indicated that the GNI series is nonstationary. Since the null hypothesis of nonstationarity is not rejected, the series needs to be transformed. After the first difference, the autocorrelation coefficients led to a conclusion that this new series is now stationary. Second differencing over the data led to overdifferenced series and was discarded from further analysis. In this way, the order of I term, *d,* in ARIMA(*p, d ,q*), has been identified (here, 1). The analysis of autocorrelation function ACF and partial autocorrelation function PACF confirmed the stationarity of differenced data set and helped in selecting the candidates for the best ARIMA model and determining whether the pattern of autocorrelation can be better explained by autoregressive AR terms, moving average MA terms or a combination of both. Selection of the best model, among few suitable, is achieved based on Akaike's Information Criterion (AIC) and Schwarz Bayesian Information Criterion (BIC). The ARIMA(0, 1, 1) model with minimal value of BIC was selected as the best, expressing the smallest error variance.

In forecasting the Serbian GNI over the period of five years (2013–2017), using the obtained ARIMA(0, 1, 1) model, values for MAE, RMSE, MSE, and MAPE were 5.98, 6.52, 42.51, and 14.92, respectively.

**Figure 7:**
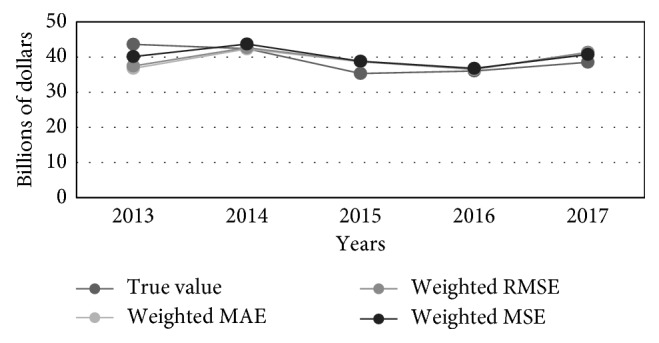
Combined ANN and linear regression models for predictions of the GNI: true, MAE-weighted, RMSE-weighted, and MSE-weighted values.

We believed that it would be interesting to extend existing experiments with additional predictions that combine ANN weights with those obtained if an appropriate weight for linear regression of the observed time series is added into a final linear combination. We have constructed a new forecasting system that exploits the performance measures of both most accurate individual ANN topologies with most accurate linear regression model. According to their performances, new weights have been calculated in order to obtain modified forecasting linear combinations of four terms. The newly obtained forecasting results are shown in Figure 7.

Corresponding performance measures in GNI forecasting in the case of linearly combined best ANN topologies and their extensions with linear regression models, outperformed the traditional ARIMA modelling. This is systematized in Table 4.

### 4.2. Prediction of the Municipal Traffic Flow

We define the traffic flow as the number of vehicles that pass a particular observation point per unit of time (usually 15 minutes). There can be various ways to “measure” the volume of the traffic and various sources of data such as simulations, sensors, taxi GPS, floating cars, and similar. In short-term prediction, which is our goal, the traffic is predicted in the next moments (usually 15 minutes) on the basis of real-time historical data.

The traffic flow time series consists of about 70 measurements, collected in collaboration with the Faculty of Transport and Traffic Engineering of the University of Belgrade, for one of the Belgrade's busiest roundabouts. This time series can also be considered as short. Here, we assume that the traffic from the immediate past has the greatest impact on the future value which, in turn, is produced for a moment in the near future. In that sense, a large series of consecutive values, we believe, can obscure the information needed for prediction. That stands especially for specific days such as state holidays (very low urban traffic) or football matches (very high urban traffic) for which the information older than several hours (at most 24 or so) has no significance. This is the reason for shortening the initial time series to 70 samples of interest.

Traffic data are accommodated and constant M (here, 140) selected so as to reduce the relative difference between output values. Although this transformation creates negative values in the training set, it is also the way to make the training process numerically better conditioned.

**Figure 8:**
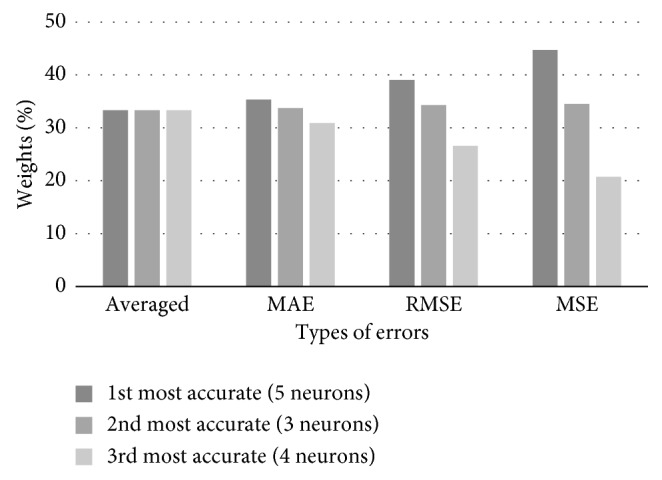
Combined weights of three best networks predicting traffic flow, corresponding to particular error types.

The initial training of 8 networks using the test set was performed. We have calculated their performance measures, i.e., MAE, RMSE, and MSE. Three most accurate FFAP networks have 5, 3, and 4 neurons in the hidden layer. Based on the values of the networks' errors, corresponding weights were calculated for the combined predictions of the test set. Combined weights of three best networks, corresponding to particular error types, are shown in Table 5, while Figure 8 gives their graphical representation.

**Figure 9:**
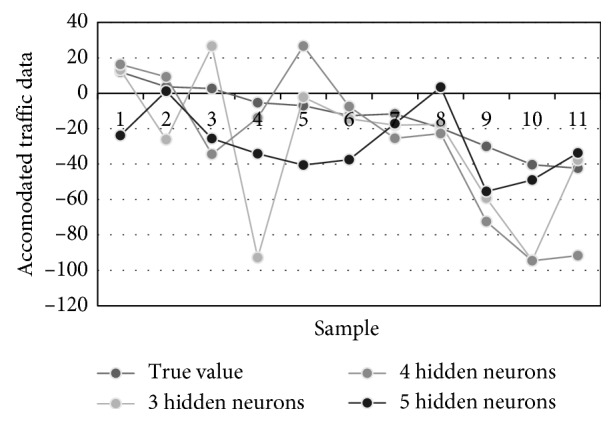
Traffic flow forecasting of three most accurate ANNs.

**Figure 10:**
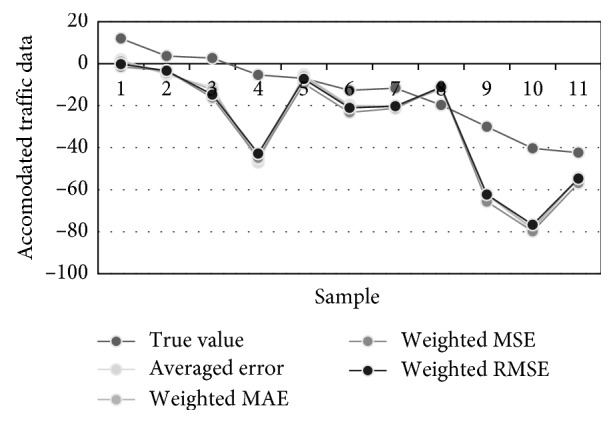
Predictions of the traffic density: true, averaged, MAE-weighted, RMSE-weighted, and MSE-weighted values.

Table 6 contains the prediction results obtained for 11 successive time instants. Previous instants were used for ANN trainings and its forecasting of the first sample in the table. This matrix also required 8 FFAP-ANNs to be trained with rising number of hidden neurons. The best performance measures are obtained for ANNs with 3, 4, and 5 neurons in the hidden layer.  Figure 9 visualizes three best traffic flow predictions from Table 6, while Figure 10 illustrates different predictions of the traffic density, i.e., true, averaged, MAE-weighted, RMSE-weighted, and MSE-weighted values.

We have again calculated errors (MAE, MSE, RMSE, and MAPE), i.e., performance measures of most accurate networks and of the concurrent, performance-based predictions for the next 11 time instances, and these are shown in Table 7. All types of errors obtained after applying our forecasting algorithm are again better than any particular FFAP. For example, the best FFAP that has 5 neurons in the hidden layer had a MAE for the entire predicted period of 20.46, while the best improvement in forecasting is achieved when RMSE-weighted concurrent linear combination of three best FFAP is applied. MAE in this case is 16.44, which is the improvement of almost 20%.

We have again performed an ARIMA fitting process in order to get the best forecasting model. The ARIMA(8,1,2) model appeared to have minimal values of AIC and BIC.

**Figure 11:**
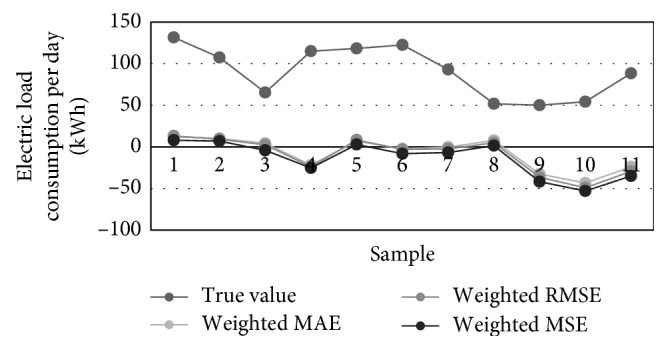
Combined ANN and linear regression models for predictions of traffic flow: true, MAE-weighted, RMSE-weighted, and MSE-weighted values.

In forecasting the municipal traffic flow for 11 samples from the test set with 95% confidence limits, using ARIMA(8,1,2) model, values obtained for MAE, RMSE, MSE, and MAPE were 47.20, 24.00, 575.83, and 389.60, respectively. Corresponding performance measures in the case of linearly combined best ANN topologies and their extensions with linear regression model performed better than the traditional ARIMA modelling, considering different types of forecasting errors. This is systematized in Table 8. Introducing the linear regression into the suggested ANN based forecasting model additionally improved the accuracy of the prediction. These predictions are shown in Figure 11.

### 4.3. Prediction of the Daily Electric Load Consumption

We define values of the electric load consumption as an average power consumed (in kWh) for a period of one day, at a particular suburban measuring point. Data for the implementation of the method are acquired from the EUNITE 2001 competition file [30]. The electric load consumption time series consists of about a hundred measurements and is considered short.

**Figure 12:**
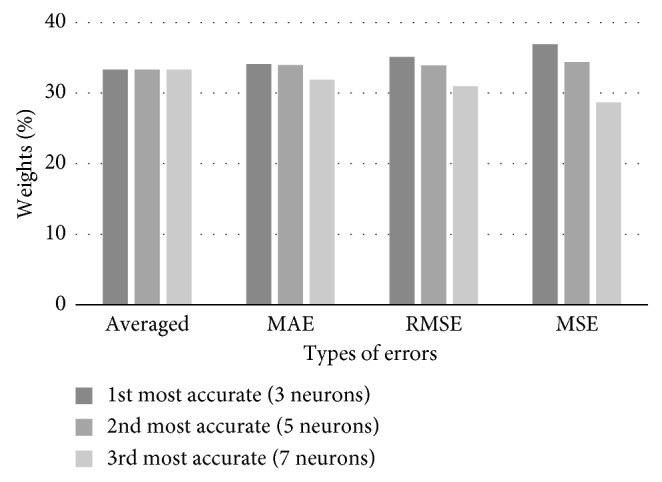
Combined weights of three best networks predicting electric load consumption, corresponding to particular error types.

Using the training set data, 8 FFAP ANNs were trained with rising number of hidden neurons. The best performance measures are obtained for ANNs with 3, 5, and 7 neurons in the hidden layer. Combined weights of three best networks, corresponding to particular error types, are listed in Table 9, while their graphical distributions are shown in Figure 12.

**Figure 13:**
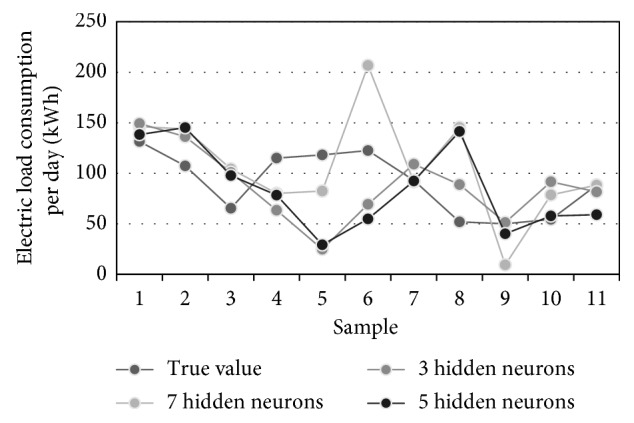
Electric load consumption forecasting of three most accurate ANNs.

**Figure 14:**
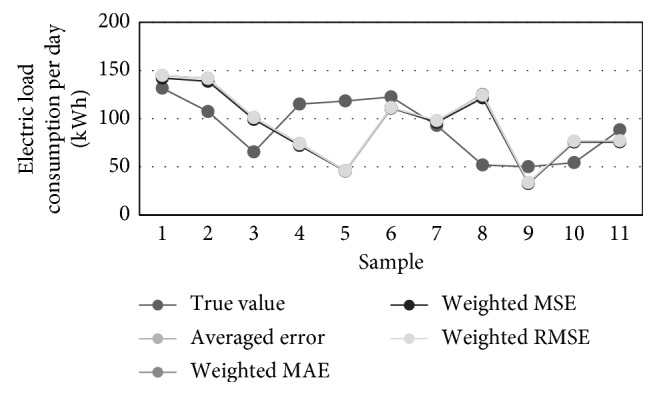
Predictions of the electric load consumption: true, averaged, MAE-weighted, RMSE-weighted, and MSE-weighted values.

Table 10 contains the prediction results obtained for 11 successive time instants of the test set.  Figure 13 visualizes three best traffic flow predictions from Table 8 while Figure 14 illustrates different predictions of the consumption, i.e., true, averaged, MAE-weighted, RMSE-weighted, and MSE-weighted values.

For this forecasting process, we have introduced additional accuracy metric, i.e., maximal error of estimate-*M* [30, 31]. It can be determined using the following equation:

(10) $$\begin{array}{c} M=\max\left(\left|y_{i}-{\hat{y}}_{i}\right|\right), \end{array}$$

where *N* again denotes the number of samples, *y*_*i*_ is the individual predicted value, and *^* ${\hat{y}}_{i}$ is the true value for the sample *i*.

Calculated errors (MAE, MSE, RMSE, MAPE, and maximal error of estimate) for prediction of the test set with 11 time instances are shown in Table 11. Errors obtained after applying our forecasting algorithm are again better than particular ANNs. For example, the best ANN that has 3 neurons in the hidden layer had a MAE for the entire predicted period of 34.42, while the best improvement in forecasting is achieved when MSE-weighted concurrent linear combination of three best ANN is applied. MSE in this case is 29.72, which is the improvement of about 13%.

We have now performed a seasonal ARIMA fitting process in order to get the best forecasting model. The SARIMA(2, 0, 2) (1, 1, 1)_7_ model had minimal BIC value.

**Figure 15:**
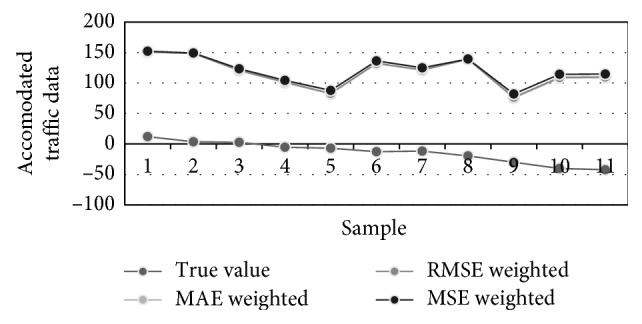
Combined ANN and linear regression models for predictions of daily electric load consumption: true, MAE-weighted, RMSE-weighted, and MSE-weighted values.

In forecasting, the municipal traffic flow for 11 samples from the test set with 95% confidence limits, using SARIMA(2, 0, 2) (1, 1, 1)_7_ model, values were obtained for MAE, RMSE, MSE, and MAPE, and maximal error of estimate were 28.27, 35.13, 1234.13, 42.38, and 85.18, respectively. Corresponding performance measures in the case of linearly combined best ANN topologies and their extensions with linear regression model did not outperform the traditional SARIMA modelling, considering different types of forecasting errors. This is systematized in Table 12. Introducing the linear regression into the suggested ANN-based forecasting model did not improve the accuracy of the prediction. These predictions are graphically represented in Figure 15.

## 5. Discussion

Results obtained using neural networks in predicting the GNI for the Republic of Serbia have multiple qualities. Specifically, the use of mathematical methods and models in the prediction of future economic trends in the Balkan region at the Southeast Euro zone, encompassing the Republic of Serbia, is very ungrateful. GNI is particularly vulnerable to turbulent changes and numerous of noneconomic factors to a great extent. Observing different types of forecasting performance measures in suggested ANN methodology just confirm the quality of the achieved results.

The results in predicting the movement of the GNI are of extreme importance for candidates for full membership in the European Union, such as Serbia. The size of the GNI determines the obligations of membership in this integration as well as the benefits that can be granted from numerous funds. The EU budget is financed by its own system of resources whose amount is limited to 1.23% of EU GNI (for the period 2014–2020). These funds are to be filled from the budged of each member country with the amount of 0.73% from its own GNI value. With the amount of about 80 billion Euros per year, these resources represent the largest source of budget revenue, reaching 69% of all revenues. On the other hand, each member can expect from the EU budget the maximal amount of 4% of its national GNI. In some situations, a small difference in the amount of the GNI can significantly change the ratio of liabilities and benefits of new members. Future GNI values are also important for creating a budget revenues and expenditures, due to restrictions in monetary and fiscal policy. Finally, the design of GNI forecasting system is crucial for making decisions on large investment projects because one gets a realistic picture of the capacity of the national economy. This reduces risks of illiquidity and even insolvency.

On the other hand, with the intention to preserve sustainable future, the importance of prediction of local traffic in large cities comes in for many reasons such as environmental and pollution monitoring; fuel usage reduction; journey planning; traffic control; urban planning; real-time route guidance; and ITS (intelligent transport system). In this analysis, ANN-based forecasting systems were developed enabling prediction of travel times, travel speeds, and traffic volumes on transportation networks using historic and real-time data.

At the end, we can also conclude that electric power load forecasting is the foundation of planning, development, and the assurance of operation efficiency and reliability of electric power systems. Because of the inherent characteristics of uncertainty, randomness, and nonlinearity, the load forecast has always been a forefront and hot issue. In the case of this forecasting task, we have achieved results using different types of forecasting performance measures in treated ANN models in order to confirm their quality.

## 6. Summary and Conclusion

In this paper, a novel methodology for increasing the predictions accuracy of different ANN-based systems has been suggested. Throughout analysis of three different time series of important everyday parameters, we have introduced some efficient improvements for prediction of short time series. The proposed method has been verified on GNI forecasting at national economy level, municipal traffic volume forecasting, and suburban daily electric load consumption forecasting. ANN-based models have been trained, and the performance of the models has been analyzed by applying various performance evaluation criteria and statistical tests that included MAE, RMSE, MSE, MAPE, and maximal error of estimate. Based on their accuracy, best performing ANN topologies, considering number of the neurons in the hidden layer, have been selected and implemented into a new forecasting system that linearly combines the forecasts of most accurate individual networks. More accurate one has been assigned a greater weight value in the linear combination. The same forecasting accuracy tests have been repeated for a test set data in order to conclude which model is superior. We have concluded that results of these three case studies reveal that linear combination of three most accurate ANN forecasts could predict trend of the future changes more accurately and with more confidence and that in most cases outperforms individual ANN forecasts, ARIMA forecasts, and hybrid ANN-linear regression forecasts. Moreover, we have determined the that accuracy improvement in these three particular cases ranges from 13% in the case of the electric load prediction and up to 65% in the case of Serbian GNI prediction. Based on the analysis presented in the paper, we can anticipate that the applicability of the method can be extended to other AI forecasting and modelling methods, as well as different types of ANN topologies. Our future research would also be oriented toward further error reductions and the discussion on the smallest number of the neurons in ANN layers in order to achieve this goal.

## Figures and Tables

**Table 1:** Forecasting weight coefficients based on the networks' training set accuracy in GNI forecasting.

Weight coefficients	7 neurons, 1^st^ most accurate	9 neurons, 2^nd^ most accurate	10 neurons, 3^rd^ most accurate
Averaged	33.33	33.33	33.33
MAE-based	40.63	31.03	28.34
RMSE-based	51.26	28.83	19.91
MSE-based	42.14	31.60	26.26

**Table 2:** GNI ANN forecasting for three most accurate FFAPs.

Year	True value	7 neurons	9 neurons	10 neurons
2013	43.64	56.45	30.17	15.42
2014	42.42	49.20	50.12	28.56
2015	35.33	38.33	42.30	34.45
2016	36.06	36.97	37.33	30.28
2017	38.55	34.73	55.25	35.57

**Table 3:** GNI forecasting errors of different ANN approaches.

Error type	7 neurons FFAP	9 neurons FFAP	10 neurons FFAP	Aver. FFAP	MAE weighted	RMSE weighted	MSE weighted
MAE	5.47	9.22	10.34	3.47	3.00	2.95	2.36
RMSE	6.85	10.68	14.36	4.78	3.70	3.20	2.60
MSE	46.92	114.02	206.23	22.84	13.68	12.27	6.77
MAPE	13.26	23.12	24.71	8.60	7.47	7.39	5.99

**Table 4:** GNI forecasting performances of different approaches.

Method	Error type			
	MAE	RMSE	MSE	MAPE
Single ANN	5.47	6.85	46.92	13.26

Combined ANN				
Averaged	3.47	4.78	22.84	8.60
MAE weighted	3.00	3.70	13.68	7.47
RMSE weighted	2.95	3.20	12.27	7.39
MSE weighted	2.36	2.60	6.77	5.99

Combined ANN-LIN.REG.				
MAE weighted	2.71	3.64	13.30	6.78
RMSE weighted	2.62	3.40	11.54	6.60
MSE weighted	2.24	2.51	6.29	5.73

ARIMA	13.26	23.12	24.71	8.60

**Table 5:** Forecasting traffic flow weight coefficients based on the networks' training set accuracy.

Weights coefficients	5 neurons, 1^st^ most accurate	3 neurons, 2^nd^ most accurate	4 neurons, 3^rd^ most accurate
Averaged	33.33	33.33	33.33
MAE based	35.34	33.73	30.93
RMSE based	39.07	34.33	26.60
MSE based	44.73	34.54	20.73

**Table 6:** Traffic ANN forecasting for three most accurate FFAPs.

Sample number	True value	3 neurons	4 neurons	5 neurons
1	12.00	13.20	16.27	−23.84
2	3.67	–26.15	9.22	1.16
3	2.67	26.61	–34.38	−25.58
4	–5.33	–92.84	–13.90	−34.12
5	–7.00	–2.21	26.67	−40.55
6	–12.67	–14.42	–7.51	−37.49
7	–11.67	–18.03	–25.44	–17.15
8	–19.67	–17.49	–22.77	3.46
9	–30.00	–59.27	–72.45	–55.44
10	–40.33	–94.37	–94.53	–49.01
11	–42.33	–37.72	–91.69	–33.72

**Table 7:** Traffic flow forecasting errors of different ANN approaches.

Error type	5 neurons FFAP	4 neurons FFAP	3 neurons FFAP	Aver. FFAP	MAE weighted	RMSE weighted	MSE weighted
MAE	20.46	23.38	22.32	16.60	16.53	16.44	18.17
RMSE	23.39	30.20	34.37	21.32	21.02	20.54	22.31
MSE	547.34	911.99	1181.01	454.45	441.86	421.84	497.88
MAPE	266.59	253.19	341.13	186.23	186.41	204.37	187.39

**Table 8:** Traffic flow forecasting performances of different approaches.

Method	Error type			
	MAE	RMSE	MSE	MAPE
Single ANN	20.46	23.39	547.34	266.59

Combined ANN				
Averaged	16.60	21.32	454.45	186.23
MAE weighted	16.53	21.02	441.86	186.41
RMSE weighted	16.44	20.54	421.84	204.37
MSE weighted	18.17	22.31	497.88	187.39

Combined ANN-LIN.REG.				
MAE weighted	10.21	13.00	168.67	102.84
RMSE weighted	10.22	12.39	153.41	98.40
MSE weighted	9.67	11.35	128.84	105.95
ARIMA	47.20	24.00	575.83	389.60

**Table 9:** Electric load consumption forecasting weight coefficients based on the networks' training set accuracy.

Weights coefficients	3 neurons, 1^st^ most accurate	5 neurons, 2^nd^ most accurate	7 neurons, 3^rd^ most accurate
Averaged	33.33	33.33	33.33
MAE based	34.12	33.99	31.89
RMSE based	35.13	33.91	30.95
MSE based	36.93	34.41	28.66

**Table 10:** Electric load consumption ANN forecasting for three most accurate FFAPs.

Sample number	True value	3 neurons	5 neurons	7 neurons
1	131.54	149.29	138.41	146.25
2	107.41	136.26	145.27	143.92
3	65.44	100.88	97.84	104.24
4	115.08	63.43	78.41	80.12
5	118.29	24.84	29.32	82.51
6	122.52	69.39	54.85	206.74
7	92.98	109.1	92.36	91.51
8	51.89	88.85	141.45	145.32
9	50.12	51.37	40.14	9.38
10	54.35	91.64	57.9	78.68
11	88.39	81.68	59.03	88.35

**Table 11:** Electric load consumption forecasting errors of different ANN approaches.

Error type	3 neurons FFAP	7 neurons FFAP	5 neurons FFAP	Aver. FFAP	MAE weighted	RMSE weighted	MSE weighted
MAE	34.42	36.82	36.68	30.67	30.55	30.53	29.72
RMSE	42.31	46.35	48.02	38.18	38.05	38.00	37.28
MSE	1789.95	2148.44	2305.90	1457.48	1448.13	1443.75	1389.59
MAPE	39.00	49.23	44.11	39.66	39.57	38.43	39.52

**Table 12:** Electric load consumption forecasting performances of different approaches.

Method	Error type				
	MAE	RMSE	MSE	MAPE	Maximal error
Single ANN	34.42	42.31	1789.95	39.00	93.45

Combined ANN					
Averaged	30.67	38.18	1457.48	39.66	73.15
MAE weighted	30.55	38.05	1448.13	39.57	72.93
RMSE weighted	30.53	38.00	1443.75	38.43	72.85
MSE weighted	29.72	37.28	1389.59	39.52	72.49

Combined ANN-LIN.REG.					
MAE weighted	35.49	41.59	1729.46	50.78	87.10
RMSE weighted	35.83	41.94	1759.12	51.42	87.45
MSE weighted	37.58	43.49	1897.06	54.26	87.63

SARIMA	28.27	35.13	1234.13	42.38	85.18

## Data Availability

Data representing the Serbian GNI time series used to support this study are obtained from World Bank National Accounts data and OECD National Accounts data files. Data sets representing daily electric load consumption are obtained from the EUNITE 2001 competition file and are cited at relevant places within the text as references [30]. The rest of the time series data representing the municipal traffic flow are available from the corresponding author upon request.
